# Aortoenteric Fistula with Digestive Contrast Material Leaking in the Aneurysmal Sac

**DOI:** 10.5334/jbsr.3686

**Published:** 2024-08-23

**Authors:** Quentin Conroux, Emmanuel Agneessens, Vincent Scavee

**Affiliations:** 1 Service de radiologie, Clinique Saint Pierre Ottignies; 2 Service de radiologie, Clinique Saint Pierre Ottignies; 3 Service de chirurgie, Clinique Saint Pierre Ottignies

**Keywords:** aortoenteric fistula, aortic surgery complications, leakage of enteric contrast material, vascular fistula, peri-prosthetic infection

## Abstract

*Teaching point:* Aortoenteric fistula, a major complication of aortic surgery, can be identified with certainty on CT scan with opacification of the intestinal tract.

## Case History

A 58-year-old male patient with an aorto-biiliac graft presented to the emergency department with diffuse back and abdominal pain. The graft had been recently placed in a context of fortuitous intraoperative discovery of aortic wall infection. The patient was currently under antibiotic treatment with vancomycin and quinolones.

**Figure 1:**
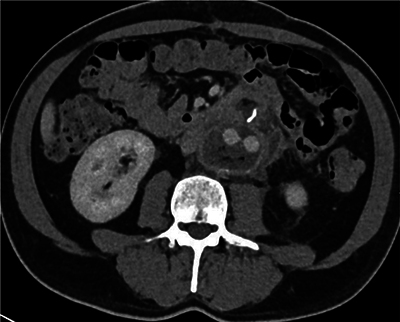
Axial CT scan showing the présence of gaz in the aneurysmal sac.

**Figure 2:**
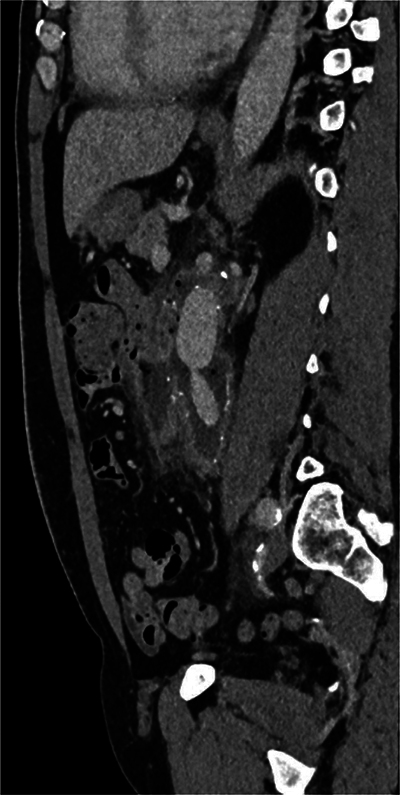
Sagital CT scan showing the close contact between a jejunal loop and the aneurysmal sac.

**Figure 3:**
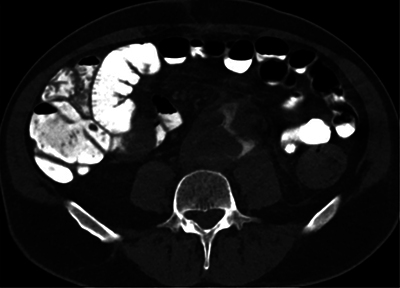
Axial CT scan showing leakage of digestive contrast into the aneurysm sac.

A laboratory workup showed increased inflammatory markers. An abdominal CT angiogram was performed. The graft was in satisfactory position, but there was an increase of gas bubbles within the aneurysm sac in comparison with the previous CT examination (2 weeks prior). A jejunal loop was in close contact with the aneurysmal sac (Figures 1 and 2). An aorto-enteric fistula was suspected. The examination was completed with a digestive opacification and delayed CT acquisition phase at 10 and 35 minutes. Digestive contrast material was progressively filling the aneurysmal cavity, confirming the diagnosis of an aorto-enteric fisula (Figure 3).

The patient was treated with an aorto-biiliac homograft, and the pathological jejunal loop was resected.

## Comments

An aorto-enteric fistula is a communication between the digestive tract (mainly the duodenum) and the aorta. Due to its poor prognosis, its identification is imperative. It is a rare complication of aortic reconstruction procedures with or without stenting. The fistula always occurs in an infectious context. As a consequence, it shares many imaging similarities with peri-prosthetic infection without fistulation. CT has a variable sensitivity (40–90%) and specificity (33–100%). Two signs are useful, especially in a context of gastrointestinal bleeding: the presence of ectopic gas and the disappearance of the fatty plane between the aorta and the digestive tract. The ectopic gas must be observed more than 4 weeks after the operation; before this period, the presence of gas could be a normal postoperative finding. Both signs are illustrated in this case. Leakage of digestive contrast material into the aneurysm sac (or vice versa), provides direct evidence of a patent fistula. However, this sign is rarely reported in the literature [1].

In conclusion, an aorto-enteric fistula is a rare and serious complication of aortic surgery. In addition, the diagnosis is challenging. The most interesting illustration of this case is the demonstration of direct communication between the aorta and the digestive tract by the leakage of contrast material from the intestinal tract, an exceptional but direct sign of a fistula.
